# The palatopharyngeal muscle in otolaryngology practice: an anatomical-based surgical report

**DOI:** 10.1007/s00405-024-08652-7

**Published:** 2024-05-02

**Authors:** Sherif M. Askar

**Affiliations:** Department of Otorhinolaryngology, Head and Neck Surgery, Faculty of Medicine, Zagazig University, Zagazig City, Zagazig, 44511 Sharkia Egypt

**Keywords:** Palatopharyngeus muscle, Sphincter pharyngoplasty, Obstructive sleep apnea

## Abstract

**Objectives:**

The author discusses current otolaryngological procedures employing the palatopharyngeus muscle, based on the surgical anatomy of the muscle and its neural supply. These techniques should be deeply revised for more conservative, anatomically-based maneuvers.

**Methods:**

Revision of anatomical and surgical research and comments with the provision of a primary concept.

**Results:**

The palatopharyngeus muscle is innervated by the pharyngeal plexus (the vagus and the accessory nerves) with additional fibers from the lesser palatine nerves. The innervation enters the muscle mainly through its lateral border.

**Conclusions:**

The palatopharyngeus muscle has a fundamental role in swallowing and speech. The muscle helps other dilators to maintain upper airway patency. Sphincter pharyngoplasty should be revised as regards its role as a sphincter. Palatopharyngeal procedures for OSA employing the palatopharyngeus muscle should follow the conservative, anatomically-based, and non-neural ablation concept.

**Level of evidence:**

4.

## Introduction

The palatopharyngeus muscle (PPM) has a pivotal role in proper swallowing and phonation. The muscle contributes to the soft palate’s movement, the pharynx’s shortening, and the hyo-laryngeal complex’s upward elevation movement. PPM is considered the key muscle in pharyngeal surgeries for obstructive sleep apnea (OSA); it has a role in the maintenance of patency of the upper airway (UA) by its activation in response to negative pressure (evident during nose breathing in the supine posture) [1–4].

The importance of PPM in Otolaryngology practice is clear; the muscle has a definite role in many surgeries, including sphincter pharyngoplsty in cases of velopharyngeal insufficiency and various pharyngeal techniques in cases of OSA. Thus, it deserves anatomical, histological, and surgical attention. However, debates still exist as regards the feasibility of various surgical techniques based on the pattern of muscle innervation.

In this commentary, the author tried to discuss some current otolaryngological procedures employing PPM, based on the surgical anatomy of the muscle. The author believes the techniques should be revised for more conservative, anatomically-based maneuvers.

## Methods

### Anatomy of the palatopharyngeal muscle

**Fig. 1:**
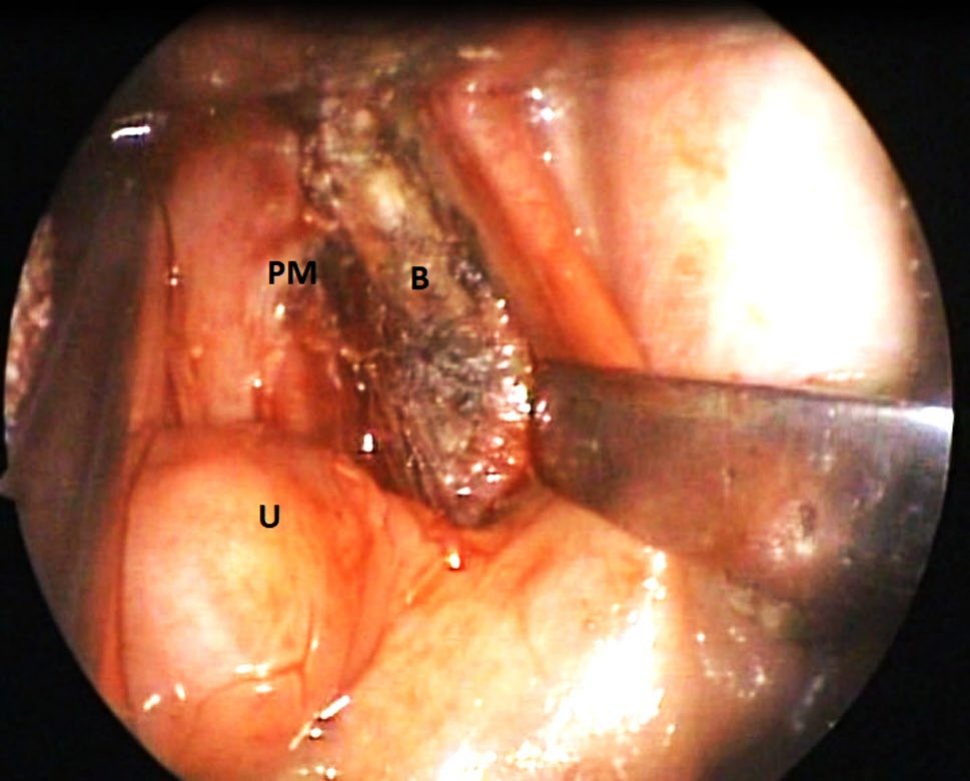
Lateral wall of the pharynx. *PM* palatopharyngeus muscle, *U* uvula, *B* bed of tonsil, the superior constrictor muscle of the pharynx

A recent cadaver head dissection reported that PPM originated from palatine aponeurosis and then passed laterally and inferiorly to form the palatopharyngeal arch (the posterior tonsillar pillar). Then, the muscle spreads radially (on the inner aspect of the pharyngeal wall) to be inserted into the pharyngeal raphe, the epiglottis, the pyriform fossa, and the thyroid cartilage. It was noticed that the most inferior portion of PPM passed antero-medially, and extended to the inner surface of the cricopharyngeal component of the inferior constrictor muscle to cover its inner surface [1, 2]. Noticeably, there is no general agreement as regards the neural supply of PPM, and debates continue. Researchers reported that the muscle receives innervation from the pharyngeal plexus (PPL). Other reports provided that the muscle is doubly innervated by fibers from the lesser palatine nerves and PPL. This innervation enters the muscle mainly through its lateral border [5–7]. The muscle predominantly consists of type II (fast twitch) fibers; thus, it is characterized by quick contraction but is more fatigable if contracted for long periods. On contraction of PPM, the soft palate moves posteriorly (with adduction of the posterior tonsillar pillars), and the lateral pharyngeal walls move medially, with elevation of the larynx. Thus, PPM could increase the velum’s size, allowing a larger area to contact the posterior pharyngeal wall [2, 8–10]. Figure 1 illustrates the anatomy of the lateral wall of the pharynx.

## Discussion

### Clinical applications

#### Swallowing

PPM was found to spread across the inner surface of the cricopharyngeal component of the inferior constrictor muscle, with dense fibrous tissue connecting them. In that way, the two PPMs could elevate the cricopharyngeal component of the inferior constrictor muscle supero-laterally, where the upper oesophageal sphincter (UES) is located. With this notice (and with the pulling forces of the suprahyoid muscles), the UES can be pulled in three different directions. Therefore, PPM might act directly on the UES [2, 8].

#### Phonation

Many authors believe that PPM acts to lower the soft palate and narrow the pharynx by the medial movement of the lateral pharyngeal walls during speech and swallowing. By its action of elevation of the larynx, the muscle could assist in the phonation of high-pitched sounds. PPM has a fundamental role in velopharyngeal closure. In addition, it has a role in the production of both oral and nasal speech sounds [1, 11–14].

By the middle of the last century, PPM was employed in cases of velopharyngeal insufficiency (VPI) in an operation called sphincter pharyngoplasty (SP). Since its presentation, SP (and its subsequent modifications) has been a cornerstone in the treatment of VPI. SP entails the creation of bilateral superiorly-based palatopharyngeal myo-mucosal flaps; flaps are transposed and sutured to the posterior pharyngeal wall or the soft palate. Postoperatively, the transposed muscle showed movements (perceived as contractions); hence the procedure was termed sphincteric pharyngoplasty. However, this assumption was not proved objectively by EMG studies and patients showed silent inactive flaps (on EMG). The noticed movements were only passive and seemed to result from the contraction of the superior pharyngeal muscles [15–18]. We assume that the primary problem (as regards SP) is that PPM is dissected from its lateral connections and is cut at its inferior ends; this means that the muscle is nearly denervated (the muscle is innervated through its lateral border) and the loss of action would be the result. So, SP would finally give only an inactive fibrous band at the velopharynx.

#### Role of PPM in OSA

Microscopic examination of PPM showed different muscle fibers in comparison with normal individuals. This change in the fibers’ properties and the disturbance of motor regulation might be important co-factors in abnormal upper airway collapse (allowing a retropalatal collapse) in patients with OSA [1–3].

The recent era of OSA surgery follows more conservative reconstructive techniques rather than aggressive radical soft tissue ablation, aiming at the preservation of pharyngeal function together with the control of OSA. PPM is involved in many palate-pharyngeal procedures and is considered a cornerstone in modern OSA surgeries. Currently, debates are still; a few authors suggest the inferior resection of PPM (superiorly-based flaps), while others favor muscle transposition without resection. Unfortunately, many basic questions would show up; could we resect PPM, how could it be resected, and how far can myofunctional therapy for OSA be effective? Although the resection of PPM might help OSA patients, it could jeopardize the beneficial functions of the muscle; therefore, PPM resection could be a “double-edged sword” in that issue. The author of the current paper follows the non-resection assumption aiming at the widening of the upper airway, with the maintenance of the muscle respiratory activities. Another important factor is that older individuals have a lower laryngeal position, which is independently a risk factor for OSA and might be associated with more severe OSA. Thus, we support the efficacy of myofunctional therapy of PPM in older individuals as it might guard against the descent of the larynx; hence, might prevent OSA in addition to its primary role in preventing dysphagia [2–5, 19–22].

### Limitations and recommendations

This report has limitations. First, it presents the author’s assumption; this assumption was based on personal and other researchers’ efforts. However, the author believes that this assumption would open discussions that would eventually contribute to a better understanding of the anatomical and surgical applications of PPM. Second, no patients were included; we think that the idea was delivered without patients’ inclusion. More multi-center research is needed for a proper understanding of that issue.

## Conclusion

The palatopharyngeus muscle has a fundamental role in swallowing and speech. The muscle helps other dilators to maintain upper airway patency. Sphincter pharyngoplasty should be revised as regards its role as a sphincter. Palatopharyngeal procedures for OSA employing the palatopharyngeus muscle should follow the conservative, anatomically-based, and non-neural ablation concept.

## Data Availability

Data is available upon reasonable request.
